# The Impact of Online Health Information on Patient Health Behaviours and Making Decisions Concerning Health

**DOI:** 10.3390/ijerph17030880

**Published:** 2020-01-31

**Authors:** Maria Magdalena Bujnowska-Fedak, Paulina Węgierek

**Affiliations:** Department of Family Medicine, Wroclaw Medical University, 51-141 Wrocław, Poland; paulina.wegierek@student.umed.wroc.pl

**Keywords:** health Internet user, online health information, e-health services, health behaviour, health decisions, impact of the Internet, patient needs, patient expectations

## Abstract

The number of Internet users searching for health-related issues increases significantly every year. The aim of this study was to investigate whether and how the information about health and disease obtained from the Internet by patients influenced them and how different e-health services can affect the patients’ choice of the doctor. The research was based on a national survey conducted among 1000 Polish adults. The study was carried out with the use of the computer-assisted telephone interviews (CATI). The study showed that e-health facilities are increasingly affecting the patient’s choice of doctor. Among the highest rated factors, the possibility of setting the date of appointment online and practice’s own website were indicated. Information on health and disease obtained from the Internet influenced respondents in many areas. Almost half of health Internet users (HI-users) wanted to change their diet and increase healthy physical activity under the influence of health information obtained online. Regarding health decision making, health information obtained from the Internet caused 45% of HI-users to make an appointment to see a doctor, and 40% of them had questions concerning diagnosis and treatment. Information on health and disease obtained from the Internet undoubtedly affects patient behaviour and health decisions they make.

## 1. Introduction

Over the last ten years, both the development of a powerful tool such as the Internet, as well as access to it have evolved very quickly. There were about 3.7 billion Internet users in late 2017 in the world. In December 2018, this number increased to 4.1 billion [1,2]. With the emergence of new opportunities, the needs of society have also changed. People use the Internet for health purposes and require access to e-health facilities more often than before.

A new type of patient appeared, the so-called e-patient, who actively gathers information about health and diseases and expects a partner relationship with the doctor [3]. Better informed and with knowledge gathered before the visit, the patients are well prepared for the visit and more often ask relevant and critical questions during the doctor's appointment [4]. On the other hand, patients often find information in unreliable sources which makes them demand inadequate diagnostics and treatment from their doctor [5]. Thus, the Internet can be either very helpful when providing essential information both for patients and doctors, but may also be misleading if it is incorrectly used [6]. Therefore, questions arise about the patients’ expectations in relation to the development of new technologies and how much they influence their health-related activities.

Nowadays, the range of the Internet is almost unlimited, which leads to the situation when the vast majority of people is using it constantly. Such enormous access to information can induce many different reactions in patients, both emotional and motivational, or lead to taking various actions by patients, crucial for diagnostic and therapeutic process. Doctors should participate in it by explaining and clarifying information acquired by patients and show them how to make the Internet a useful and reliable tool [7]. 

The possibility of contacting your doctor via e-mail or the Internet, and whether the health care provider has its own website seems to have an increasing impact on the choice of the doctor by the patient. Thanks to access to their own medical records and facilitating doctor-patient communication, the engagement of patients in the therapeutic process can improve [8]. There are more and more studies on the impact of the Internet on the patients’ behaviour and health decisions, but the data are still ambiguous and insufficient [6,9,10]. Therefore, the aim of the study was to investigate whether and how the information about health and disease obtained from the Internet by patients influenced them and how different e-health services can affect the patients’ choice of the doctor. One of the most important goals of the study was to determine how important in choosing a doctor is access to information about practice and the possibility of contact by e-health facilities with health care professionals. Another essential issue was the impact of the Internet on the patient. The study of the impact of online health information covered three areas of life: basic emotions, such as anxiety and fear, as well as relief and support, health intentions, such as the desire to change lifestyle and habits, as well as self-reported health decisions, including medical consultation, taking or resignation from testing or treatment. 

## 2. Materials and Methods 

### 2.1. Study Design

The research was based on a survey conducted among Polish adults from December 2017 to January 2018. The sample collection was carried out by the national opinion poll agency (Kantar TNS), with the use of the computer-assisted telephone interviews (CATI). The work of interviewers in the CATI studio took place under the control of experienced supervisors and the course of the study was monitored on a current basis. Both landline and mobile numbers were called with the penetration amounting to 13% and 90% respectively. During the selection of telephone numbers, the equal distribution of respondents in terms of voivodships, size of city, age, and sex was taken into account. The average response rate was 5.2%. The non-response group included invalid phone numbers, failure to answer the phone and refusal to take part in the research. In case of any above situations, the telephone number was replaced with another one with the same characteristics. Questionnaires were collected until a representative research group of 1000 people was obtained.

All subjects gave their informed consent for inclusion before they participated in the study. The study was conducted in accordance with the Declaration of Helsinki, and the protocol was approved by the Ethics Committee of Wroclaw Medical University (ST C290.17.040).

### 2.2. Questionnaire

The study was part of the national survey prepared on the basis of our previous studies concerning trends and patterns of health-related Internet use [11,12,13,14]. The survey contained 22 original questions in Polish. Due to language uniformity in the country, no other language versions were needed. This study focused on two complex questions. 

In the first complex question, the respondents were asked whether factors related to online services would play a role in the decision concerning the choice of a new doctor. The question concerned information about medical practice in the Internet and on-line access to medical records. Respondents were also asked about the significance of the possibility to book or reschedule visits via the Internet. Subsequently, respondents were questioned about the importance of on-line interactivity, like the possibility of receiving simple medical recommendations via text messages or e-mails, or prescribing medications electronically. The questions were answered according to Likert scale from 1 to 5 (where 1 means unimportant and 5 stands for very important), with an additional option ‘I do not know/not applicable’.

The next analyzed question was about the consequences of information about health or illnesses obtained from the Internet in three main fields: basic emotions, health intentions and self-reported health decisions. In term of emotions, the questions concerned the feeling of anxiety or fear, as well as comfort or relief triggered by information obtained from the Internet. As far as intention is concerned, respondents were asked about the desire to change a diet, the willingness to change other habits resulting from lifestyle, e.g., smoking cigarettes or alcohol consumption, and the need to increase physical activity. When it comes to self-reported health decisions, the questions concerned making suggestions or questioning a doctor about the diagnosis or treatment, requesting a referral for additional screening tests, taking or changing the medication without consulting a doctor, making an appointment or cancelling the doctor’s or other health care provider’s appointment. For each of these questions there was a possibility to answer ‘yes’, ‘no’, or ‘ I don’t know’. 

Additional demographic data of respondents, such as sex, age, education, the size of the city or professional situation, were also obtained. The whole questionnaire is available in Supplementary Materials.

### 2.3. Data Analysis

Three sociodemographic groups were distinguished among respondents: all respondents, Internet users (I-users) and the Internet users searching for health-related issues (HI-users). The I-users group included everyone who used the Internet or asked others to do so for the respondent. HI-users were defined as all respondents who searched for health information on the Internet, even if they did it less than once a year. Defined, the three groups of respondents (all respondents, I-users, HI-users) are not disjointed, therefore it was impossible to assess by standard methods statistical significance of differences between them. However, it was possible to assess separately in each of these groups, for example, whether there were differences between the categories of the studied variable. A descriptive analysis of distinguished groups, followed by a statistical analysis of HI-users, was carried out in order to identify significant associations between the participant independent variables and influence of online health information on basic emotions, health intentions and self-reported health decisions. The statistical package R software (version 3.5.2, R Foundation for Statistical Computing, Vienna, Austria) was used in the calculations. Normality of distributions of quantitative variables was checked by the Shapiro–Wilk test. None of the variables had a normal distribution, therefore in the further analysis, non-parametric ones were used, such as: exact Fisher’s independence test and Chi-square test for equal proportions (homogeneity test). The significance level of 0.05 was assumed in all tests. 

## 3. Results

### 3.1. Characteristic of Respondents

The study group consisted of 1000 people, including 558 women and 442 men. The median age of respondents was 53 (min-max: 18–88). Among the study group, 887 people declared that they used the Internet; 86.6% of them used it for health purposes. 95.7% of respondents used cell phones. More information about the study population is provided in Table 1.

### 3.2. Key Factors Affecting the Choice of the Doctor

E-health facilities were important for patients making a decision related to choosing a new doctor. In the respondents’ opinion, 75% of e-health facilities were assessed as at least quite important. HI-users appreciated the e-health services more than all respondents, however, the differences were small, which is suggested by median and inseparable ranges of mean+/_ SD (see Table 2).

According to HI-users, among the highest rated factors were the possibility of making an appointment or changing the date of the visit via Internet and if the practice had its own website. Information about practice’s certificates in the Internet and the possibility to receive SMS (short message service) notifications about the upcoming appointment were rated not much lower. Respondents recognized the possibility of receiving simple medical recommendations and the possibility to prescribe medications electronically as the least influencing, see Table 2.

### 3.3. Emotions Triggered by Health or Disease Information Obtained From the Internet

Information obtained from the Internet can affect patients both positively and negatively. Almost one third of respondents felt anxious or experienced fear and one fourth felt relieved (see Table 3). Feeling support or relief depended on sex, residency type and frequency of Internet use. Women (27.2%) and those having a roommate (25.2%) more often experienced relief than men (19.9%) and those living alone (15.1%). Along with the increase in the frequency of using the Internet, the growth of feeling of relief was also observed. For comparison, 39.4% of those using the Internet every day felt relieved or supported, while only 15.2% of those who used the Internet at least once a year felt such emotions.

On the other hand, the presence of feelings of anxiety or fear depended on sex and education. In women (35.3%), the influence of the information obtained on the Internet was more often observed than in men (22.6%). Better educated respondents are more often affected by feelings of anxiety and fear caused by online information, for example 34.5% of respondents with higher or some higher and only 21.0% of respondents with basic or vocational education had that feelings, see Table 4. For the clarity of data, some statistically insignificant variables have been omitted in the table. The table with full data is provided in Supplementary Materials.

### 3.4. Health Intentions and Self-Reported Pro-Health Behaviour Caused by Health or Disease Information Obtained From the Internet

Almost half of HI-users wanted to change their diet and to increase healthy physical activity under the influence of information obtained from the Internet. Much less, because only 19.5% of them wanted to change their habits, e.g., quit smoking, or limit the consumption of alcohol, see Table 3. 

The desire to change the diet due to information about health obtained from the Internet depended on age, sex, education and the frequency of the Internet use. The youngest respondents (aged 18–35) (55.3%) and women (52.2%) more often showed willingness to change their diet than older people (47.1% for aged 36–59 and 39.4% for respondents over 60 respectively) and men (44.2%). This desire was also growing along with the level of education (basic/vocational 41.9%, secondary 47.8%, higher/some higher 55.2% respectively) and frequency of using the Internet for health purposes (72.7 % for respondents using Internet daily and only 22.9% for those searching online less than once a year).

In turn, the desire to change other habits resulting from the lifestyle, such as quitting smoking or limiting alcohol consumption depended on age, sex, residency type and employment status. Middle (16.7%) and older-aged (16.3%) people less often showed willingness to change other lifestyle habits than younger people (24.5%). Unlike the desire to change their diet, men (25.7%) quit smoking or reduced their alcohol consumption more often than women (14.5%). Students (39.0%) and people living in urban areas (22.2%) were more willing to change their habits than other occupational groups and residents of rural areas (14.4%). 

Increasing healthy physical activity, reported by respondents, triggered by information obtained from the Internet depended on age, education, residency place, employment status, frequency of Internet use and health status. Younger, better educated and living in urban areas people more often increased physical activity. As the frequency of using the Internet for health purposes increased, the frequency of physical activity was higher as well. On-line information about healthy physical activity affected to a greater extent respondents assessing their health as good or very good (53.0%) than those evaluating their health status as poor or very poor (28.9%), see Table 5. For the clarity of data, some statistically insignificant variables have been omitted in the table. The table with full data is attached in Supplementary Materials.

### 3.5. Self-Reported Health Decisions Caused by Health or Disease Information Obtained From the Internet

Information obtained from the Internet most often caused making an appointment to see a doctor. It concerned 45% of the Internet users searching for health-related issues. Further, 40% of HI-users had suggestions and questions about treatment or diagnosis and 35% of them asked for referral to make screening or additional tests due to information obtained from the Internet. They relatively rarely decided to cancel or postpone the visit (22% of HI-users), even less often they gave up planned tests (6.8% of HI-users) or took or changed medication without consulting their doctor (8.7% of HI-users), see Table 3.

Making an appointment to see a doctor depended on education, place of residence and the frequency of Internet use for health purposes. With the increase in the level of education and the regularity of using medical Internet websites, the frequency of making an appointment to see a doctor increased. Respondents living in the urban area (47.7%) more often made an appointment to see a doctor than the rural area’s residents (40.1%). Furthermore, making suggestions and asking questions of a doctor were significantly related to Internet use and increased along with the growth of Internet use for health purposes.

Making a decision to resign from planned tests or used medications were significantly related to the frequency of Internet use and using a mobile phone. It is interesting that with the increase in the frequency of Internet use, the number of patients giving up tests or medications was higher. However, mobile phone users (6.5%) made such decisions less often than people who do not use mobile phones (26.7%).

Cancelling or changing the date of a medical appointment depended on employment status and the frequency of using the Internet for health purposes. With the increase in the frequency of Internet use for health purposes, the number of patients cancelling or changing the date of a doctor’s appointment was greater than before. The unemployed (37.5%) resigned from visits most often, the least often—the retired and permanently incapable (15.7%), see Table 6.

There were no characteristic features associated with taking the medication or changing it without consulting the doctor and information obtained from the Internet. For clarity of data, some statistically insignificant variables have been omitted in the Table 6. The table with full data is attached in Supplementary Materials.

## 4. Discussion

A technological revolution has taken place in recent years, especially in terms of the Internet access and, consequently, access to online health-information. The Internet has become a source of quickly and easily accessible information that patients are increasingly willing to use. Information, in turn, evokes a lot of emotions, motivations, and is important in making decisions concerning health, also when choosing a new doctor.

A survey conducted in 2018 by Accenture among US residents showed that for 75% of interviewees the healthcare technology was at least important to some extent. Technologies used by patients to deal with the health issues included most often websites (56%) and mobile phones or tablets (46%). Electronic health records (38%) and social media (35%) were also meaningful [15]. This data correspond to our findings, in which 76.8% of respondents declared using the Internet for health purposes. According to our study, there are several e-health facilities which play an important role while choosing a new doctor. The most important ones are doctor’s own website and information about their medical practice, such as its position with respect to other practices.

For many years, customers have been searching for service providers online and checking how other Internet users rate them before making a decision. These trends have also spread to the area of health care services. They already play an important role in providing information that influences the decision to choose the right doctor and their impact is likely to increase in the future [16,17]. 

The impact of e-health facilities could be different in various countries due to disparate healthcare systems. When it comes to private healthcare, it has rules of the free market and it is clear that the client, in this case the patient, chooses freely a healthcare provider. It looks differently in the public healthcare system, where the choice of a doctor or institution is often limited. In Poland, the public healthcare system enables the patient to choose a family physician. In case of a need of out-patient specialist treatment, a referral of a doctor who practices within primary health care system is required, except for oncologist, gynecologist and obstetrician, dermatologist and psychiatrist. This model is popular in many other European countries, such as Great Britain, Denmark, or Finland, where the family doctor has a gate keeper function for other specialists [18,19,20]. Another model is the total freedom of choice of a family doctor and other specialists, without referral needed, where a patient can arrange any subsequent appointment to other doctor, both to continue treatment and to look for a second opinion. Such a system works, e.g., in Belgium and Luxemburg [21,22].

According to recent studies, 35% of respondents have searched for online doctor reviews over the past years. The study also showed that these online reviews had a great influence on selecting a doctor: among those who searched for information about online doctor ratings, 35% reported choosing a doctor based on positive ratings, and 37% admitted avoiding a doctor with negative ratings [23]. These data are consistent with our research. However, it remains unclear if online assessments can reflect the quality of medical care offered by individual healthcare providers [16,17]. Moreover, it should be noted that online assessments do not always coincide with the actual effectiveness of evaluated doctors. In a study of cardiac surgeons practicing in the USA who publicly report their results, there was no correlation found between the online assessment of a doctor and risk adjusted mortality rates [24].

Our study revealed that the possibility of making an appointment or changing its date via the Internet is an important key factor affecting the choice of a doctor. Surprisingly, in Australian study conducted in 2015, 29 months after an e-appointment service had been introduced, only 4% of patients of primary care clinic used it. For the rest of them, calling a clinic was an easier way of making an appointment and they also preferred verbal communication with receptionists [25]. The interest of our patients can be explained by the period of time which passed between studies. In the last few years, there has been a significant technological development and the availability of online services in various spheres of life has considerably increased.

As our study showed, a possibility to prescribe medications via the Internet had a little meaning when choosing a new doctor. In Poland, e-prescriptions are still being introduced, as the next step after introducing obligatory electronic sick leave and are not popular as yet. This is a possible reason why they were low rated by patients. Over the world, nationwide e-medication systems are recognized as important objectives and are constantly being developed and improved. However, this process is slow, mainly due to privacy and security requirements and the need for changes in legal regulations, which also applies to Poland [26,27].

Health and disease information obtained from the Internet has a significant influence on patients’ emotions, intentions, and self-reported behaviours. When it comes to emotions triggered by health information obtained from the Internet, feelings of anxiety or fear were reported by a similar number of respondents in 2015 [14] and in 2018 (31.0% and 29.6%, respectively). At the same time, the number of respondents feeling relief or support decreased from 31.3% in 2015 [14] to 24.1% in 2018. A Dutch study indicated that health anxiety is related to an increase in online health information searches. Individuals who are more concerned about their health also experience more negative effects from online health information search [28].

Online health information also caused changes in intentions and self-reported pro-health behaviours of HI-users. Our results are very similar to those obtained in 2015, especially as regards willingness to diet change (48.0% in 2015, 48.8% in 2018, respectively) [14]. A percentage of respondents who wanted to change habits such as quitting smoking or limiting alcohol consumption was relatively small and amounted to 19.5%. The Internet remains an underestimated source of smoking cessation interventions. Appropriate online advertising creates the chance to recruit more smokers to cessation interventions than traditional methods. Moreover, it gives the opportunity for a better evaluation of cessation progress [29].

Our research has shown persistent trends in health decision making due to information obtained on the Internet. Results proved to be comparable to the outcomes from 2015 [14]. Similar proportion of patients was asking for suggestions or making queries concerning the diagnosis and treatment during medical visit (46% in 2015, 40.1% in 2018). These statistics also apply to the negative effects of information obtained from the Internet, such as cancellation of a doctor’s appointment (20.4% in 2015, 21.9% in 2018) and changing the medications use without consulting the doctor (7.7% in 2015, 8.7% in 2018) but they fortunately remain low [14]. Over the last 10 years, the number of people who have decided to make an appointment to see a doctor due to health information obtained from the Internet has increased from 38.3% in 2007 [4] to 45% in 2018, according to our study. A recent Australian study on emergency department (ED) showed that patients very often checked their health information regarding their current problem before presenting to ED. It was surprising that online information had a positive impact on the doctor–patient interaction. Even due to conflicting online sources, most patients would never or rarely doubt their diagnosis (79%), or change their treatment plan (91%) [30].

It turns out that certain features predispose to some health-related behaviours arising under the influence of the Internet [12,31]. Women used the Internet more often for health purposes (56.2%). They also reacted more emotionally due to information about health and disease obtained from the Internet than men and more frequently wanted to change their diet. Many studies show that women more often search for health information on the Internet. They are also interested in health information to a greater extent and more often seek active health-related information than men [32,33].

In turn, younger respondents were more willing to change their diet, habits, and increase physical activity than older ones. According to other studies, approximately 80% of young adults trust online information and consider the Internet to be an important source of health information [34]. Italian research has also confirmed that both women and younger people use the Internet more often for health purposes [35].

Relationship between residency type and HI-users emotions and self-reported behaviours turned out to be interesting. Respondents living alone get more support and relief from the Internet than those having a roommate, while there is no such relationship when it comes to anxiety and fear. Furthermore, people living with someone are more motivated to increase physical activity than those living alone. A systematic review of 36 publications showed that loneliness can negatively affect starting some physical activity, what is in accordance with our findings. It is worth mentioning that at the same time it was noted that physical activity reduces loneliness [36].

It seems that education is another strong factor affecting the approach to information obtained from the Internet. Our study showed that less educated people more often felt anxious or even frightened by online health information. It was also easier to affect that group in a positive way, encouraging them to change a diet or to increase healthy physical activity. Moreover, better educated respondents less often made an appointment to see a doctor due to information obtained from the Internet. However, there is still too little data on this subject and the relationship requires confirmation obtained in the course of other studies.

The HI user group included people using the Internet for health purposes at different frequencies. Our research has confirmed that people who use the Internet more often are more affected by it. This relationship concerned both emotions and self-reported health behaviours and health decisions.

The study has several limitations. The response rate was low (5.2% on average). This may be due to failure to answer a phone from unknown callers, the lack of time and widespread rush that prevents a relatively long interview. The date when the research was conducted was not without significance, as it took place during the holiday break (December–January). Many respondents may also have difficulty in deliberately answering questions because of the high speed at which phone calls are usually made. With the development of modern information and communication technologies, electronic forms where respondents have the opportunity to reflect on the answer are becoming increasingly popular. Telephone surveys, however, have their undoubted advantages, such as the ability to clarify a question if the respondent does not understand its content, which is impossible in the case of electronic surveys. In addition, unfortunately, it is not known how the desire to change diet and other habits affected the actual actions of the respondents. Likewise, it is not known to what extent the subjects who declared healthy physical activity actually increased it. Exploring this topic, however, is material for separate research.

## 5. Conclusions

Health and disease information obtained from the Internet undoubtedly influence patients’ behaviour and decisions relating to health. Along with the popularisation of the Internet, the demand for e-health services is also increasing. The e-health facilities such as the doctor’s office’s own website and the possibility of making an appointment or changing its date via the Internet are important for patients, especially when choosing a new doctor. Among self-reported pro-health behaviours and health decisions, patients are most often willing to change their diet and increase healthy physical activity under the influence of the Internet. They are also more often making an appointment to see a doctor and asking questions about the diagnosis or treatment of certain diseases. Women are more likely to be affected by the Internet. Younger, better educated people living in the city more often take into account information obtained from the Internet as well. There is certainly a need to continue studies in this field. It seems to be necessary to follow the needs of the Internet users searching for health-related issues in order to make the diagnostic and therapeutic process as effective and satisfying as possible for both doctors and patients.

## Figures and Tables

**Table 1:** Characteristics of the study group.

Characteristics		All Respondents (*n* = 1000)		I-Users (*n* = 887)		HI-Users (*n* = 768)	
		*n*	%	*n*	%	*n*	%
Age groups	18–35	302	30.2	292	32.9	274	35.6
	36–59	429	42.9	411	46.3	362	47.1
	60+	269	26.9	184	20.7	133	17.3
Sex	Female	558	55.8	486	54.8	432	56.2
	Male	442	44.2	401	45.2	337	43.8
Education	Basic/Vocational	339	33.9	265	29.9	210	27.3
	Secondary	373	37.3	342	38.6	297	38.6
	Higher/Some higher	288	28.8	280	31.6	262	34.1
Residency type	Alone	143	14.3	112	12.6	88	11.5
	With family/ Other	856	85.7	774	87.4	680	88.5
Residency place	Rural	387	38.7	329	37.1	273	35.5
	Urban	613	61.3	558	62.9	496	64.5
Employment status	Education	44	4.4	44	5	41	5.3
	Paid work/Voluntary/Other	594	59.4	577	65.1	522	67.9
	Retired/Permanently sick or disabled	317	31.7	223	25.1	166	21.6
	Unemployment	45	4.5	43	4.8	40	5.2
Health status	Good/Very good	570	57.5	547	62.2	495	64.8
	Fair	347	35	279	31.7	232	30.4
	Poor/Very poor	74	7.5	54	6.1	37	4.8
Mobile phone use	Yes	957	95.7	861	97.1	754	98
	No	43	4.3	26	2.9	15	2
Frequency of Internet use for health purposes	Daily	33	3.7	33	3.7	33	4.3
	At least once a month	443	49.8	443	49.9	443	57.6
	At least once a year	245	27.5	245	27.6	245	31.9
	Less than once a year	48	5.4	48	5.4	48	6.2
	Never	121	13.6	118	13.3	0	0

**Table 2:** Key factors affecting the choice of the doctor.

Key Factors Affecting the Choice of the Doctor	All Respondents	Rank	I-Users	Rank	HI-Users	Rank
	Mean±SD Median		Mean±SD Median		Mean±SD Median	
The possibility of receiving simple medical recommendations via SMS or e-mail	2.79 ± 1.55 3	7	2.81 ± 1.53 3	6	2.86 ± 1.52 3	7
The possibility to prescribe medicines via e-mail or the Internet	2.66 ± 1.65 2	8	2.77 ± 1.64 3	7	2.89 ± 1.63 3	6
The possibility of setting or changing the date of the visit via the Internet	3.44 ± 1.65 4	3	3.62 ± 1.57 4	1	3.72 ± 1.53 4	1
Information about medical practice on the Internet (certificates, practice position among other medical facilities)	3.4 ± 1.55 4	4	3.55 ± 1.48 4	3	3.67 ± 1.42 4	2
The doctor’s office has its own website	3.51 ± 1.52 4	2	3.62 ± 1.45 4	1	3.72 ± 1.39 4	1
The possibility of contact via e-mail	3.09 ± 1.6 3	6	3.23 ± 1.56 4	5	3.36 ± 1.51 4	5
The possibility to receive SMS notifications	3.55 ± 1.5 4	1	3.6 ± 1.45 4	2	3.66 ± 1.41 4	3
Online access to the patient’s medical records	3.37 ± 1.67 4	5	3.53 ± 1.6 4	4	3.64 ± 1.55 4	4

**Table 3:** Emotions, health intentions and self-reported health decisions caused by online health-related information *.

	HI-users						
	yes		no				
	*n*	%	*n*	%	Chi2	df	*P* **
Feeling anxious or fear	226	29.6	537	70.4	126.76	1	**0**
Feeling of support or relief	184	24.1	580	75.9	205.26	1	**0**
Desire to change diet	373	48.8	392	51.2	0.470	1	0.492
Desire to change other habits, ex. quitting smoking, limiting alcohol	184	19.5	612	80.5	283.28	1	**0**
Increasing healthy physical activity	347	45.5	416	54.5	6.240	1	**0.012**
Asking the doctor for suggestions or questions about the diagnosis or treatment of diseases	306	40.1	458	59.9	30.24	1	**0**
Asking the doctor for referral / additional screening tests	266	34.9	497	65.1	69.94	1	**0**
Taking the drug or changing the medication without consulting a doctor	67	8.7	700	91.3	522.41	1	**0**
Resignation from planned tests or medicines used	52	6.8	712	93.2	570.16	1	**0**
Making an appointment with a doctor	345	45	421	55	7.54	1	**0.006**
Cancellation of a doctor's appointment	168	21.9	599	78.1	242.19	1	**0**

* Significant differences between positive and negative responses for selected HI-users reactions are marked in bold; ** Calculated statistical significance in the Chi-square homogeneity test.

**Table 4:** Emotions triggered by health or disease information obtained from the Internet*.

Characteristics	Feeling Anxious or Fear					Feeling of Support or Relief				
	yes		no		*p*	yes		no		*P* **
	*n*	%	*n*	%		*n*	%	*n*	%	
Sex					**0**					**0.021**
Women	152	35.3	278	64.7		117	27.2	313	72.8	
Men	76	22.6	260	77.4		67	19.9	269	80.1	
Education					**0.003**					0.527
Basic/Vocational	44	21.0	166	79.0		44	21.2	164	78.8	
Secondary	94	31.9	201	68.1		74	25.0	222	75.0	
Higher/Some higher	90	34.5	171	65.5		66	25.2	196	74.8	
Residency type					1					**0.044**
Alone	25	29.1	61	70.9		13	15.1	73	84.9	
With family/Other	203	29.9	476	70.1		171	25.2	508	74.8	
Mobile use					1					**0.05**
Yes	224	29.8	527	70.2		177	23.5	575	76.5	
No	4	26.7	11	73.3		7	50.0	7	50.0	
Frequency of Internet use for health purposes					0.051					0
Daily	12	36.4	21	63.6		13	39.4	20	60.6	
At least once a month	145	32.9	296	67.1		127	28.9	312	71.1	
At least once a year	59	24.4	183	75.6		37	15.2	207	84.8	
Less than once a year	10	21.3	37	78.7		7	14.6	41	85.4	

* Significant differences between positive and negative responses for selected HI-users reactions are marked in bold; ** Calculated statistical significance in Fisher’s exact independence test.

**Table 5:** Self-reported pro-health behavior triggered by health or disease information obtained from the Internet *.

	The Desire to Change Diet					The Desire to Change Other Habits, ex. Quitting Smoking, Limiting Alcohol					Increasing Healthy Physical Activity				
Characteristics	yes		no		*P* **	yes		no		*P* **	yes		no		*P* **
	*n*	%	*n*	%		*n*	%	*n*	%		*n*	%	*n*	%	
Age groups					**0.008**					**0.034**					**0**
18–35	152	55.3	123	44.7		67	24.5	207	75.5		151	54.9	124	45.1	
36–59	170	47.1	191	52.9		60	16.7	300	83.3		167	46.5	192	53.5	
60+	52	39.4	80	60.6		21	16.3	108	83.7		31	23.3	102	76.7	
Sex					**0.029**					**0**					0.189
Women	225	52.2	206	47.8		62	14.5	366	85.5		187	43.4	244	56.6	
Men	149	44.2	188	55.8		86	25.7	249	74.3		162	48.2	174	51.8	
Education					**0.015**					0.456					**0**
Basic/ Vocational	88	41.9	122	58.1		36	17.4	171	82.6		73	34.8	137	65.2	
Secondary	142	47.8	155	52.2		55	18.6	240	81.4		139	47.0	157	53.0	
Higher/ Some higher	144	55.2	117	44.8		57	21.8	204	78.2		137	52.5	124	47.5	
Employment status					0.093					**0.015**					**0**
Education	22	53.7	19	46.3		16	39.0	25	61.0		27	65.9	14	34.1	
Paid work/ Voluntary/ Other	260	49.8	262	50.2		96	18.4	425	81.6		255	49.0	265	51.0	
Retired/ Permanently sick or disabled	68	41.2	97	58.8		27	16.7	135	83.3		46	27.7	120	72.3	
Unemployment	24	60.0	16	40.0		9	23.1	30	76.9		21	52.5	19	47.5	
Residency type					0.734					0.667					**0.026**
Alone	41	46.6	47	53.4		18	20.9	68	79.1		46	51.7	43	48.3	
With family/Other	333	49.0	346	51.0		130	19.2	546	80.8		303	44.8	374	55.2	
Residency place					0.087					**0.028**					**0.026**
Rural	120	44.0	153	56.0		39	14.4	232	85.6		106	39.0	166	61.0	
Urban	254	51.3	241	48.7		109	22.2	383	77.8		243	49.1	252	50.9	
Health status					0.052					0.532					**0**
Good/Very good	256	51.8	238	48.2		94	19.1	397	80.9		260	53.0	231	47.0	
Fair	102	44.2	129	55.8		44	19.2	185	80.8		77	33.0	156	67.0	
Poor/Very poor	14	36.8	24	63.2		10	26.3	28	73.7		11	28.9	27	71.1	
Frequency of Internet use for health purposes					0					0.481					**0.009**
Daily	24	72.7	9	27.3		8	24.2	25	75.8		19	57.6	14	42.4	
At least once a month	235	53.3	206	46.7		90	20.5	348	79.5		216	49.1	224	50.9	
At least once a year	103	42.4	140	57.6		44	18.3	197	81.7		98	40.3	145	59.7	
Less than once a year	11	22.9	37	77.1		6	12.5	42	87.5		14	29.8	33	70.2	

* Significant differences between positive and negative responses for selected HI-users reactions are marked in bold; ** Calculated statistical significance in Fisher’s exact independence test.

**Table 6:** Self-reported health decisions triggered by health or disease information obtained from the Internet*.

Characteristics	Making an Appointment with a Doctor					Cancellation of a Doctor’s Appointment					Resignation from Planned Tests or Medicines Used				
	yes		no		*P* **	yes		no		*P* **	yes		no		*P* *
	*n*	%	*n*	%		*n*	%	*n*	%		*n*	%	*n*	%	
Sex					0.771					0.539					0.085
Women	196	45.5	235	54.5		98	22.7	334	77.3		36	8.4	395	91.6	
Men	150	44.4	188	55.6		70	20.7	268	79.3		17	5.1	319	94.9	
Education					**0.007**					0.2					0.321
Basic/vocational	81	38.8	128	61.2		49	23.3	161	76.7		19	9.1	189	90.9	
Secondary	127	42.8	170	57.2		55	18.5	242	81.5		17	5.7	280	94.3	
Higher/some higher	138	52.5	125	47.5		64	24.3	199	75.7		17	6.5	245	93.5	
Employment status					0.25					**0.022**					0.691
Education	17	41.5	24	58.5		10	24.4	31	75.6		4	9.8	37	90.2	
Paid work/ voluntary/ other	248	47.4	275	52.6		117	22.4	406	77.6		37	7.1	483	92.9	
Retired/ permanently sick or disabled	64	38.8	101	61.2		26	15.7	140	84.3		9	5.4	157	94.6	
Unemployment	17	42.5	23	57.5		15	37.5	25	62.5		3	7.5	37	92.5	
Residency place					**0.05**					0.262					0.806
Rural	109	40.1	163	59.9		52	19.0	221	81.0		21	7.7	251	92.3	
Urban	237	47.7	260	52.3		116	23.3	381	76.7		32	6.5	463	93.5	
Mobile use					1					0.109					**0.016**
Yes	339	45.0	415	55.0		162	21.5	593	78.5		49	6.5	703	93.5	
No	7	46.7	8	53.3		6	40.0	9	60.0		4	26.7	11	73.3	
Frequency of Internet use for health purposes					**0.007**					**0.02**					**0.005**
Daily	19	57.6	14	42.4		12	36.4	21	63.6		5	15.6	27	84.4	
At least once a month	215	48.6	227	51.4		107	24.2	336	75.8		37	8.4	404	91.6	
At least once a year	97	39.9	146	60.1		40	16.5	203	83.5		7	2.9	236	97.1	
Less than once a year	14	29.2	34	70.8		9	18.8	39	81.2		3	6.2	45	93.8	

* Significant differences between positive and negative responses for selected HI-users reactions are marked in bold; ** Calculated statistical significance in Fisher’s exact independence test.

## Supplementary Materials

The following are available online at https://www.mdpi.com/1660-4601/17/3/880/s1, File S1: E-Health consumer trend survey 2017, Table S1: Emotions triggered by health or disease information obtained from the Internet*, Table S2: Self-reported pro-health behavior triggered by health or disease information obtained from the Internet*, Table S3: Self- reported health decisions triggered by health or disease information obtained from the Internet*.
