# Supplementary material for: The Impact of Online Health Information on Patient Health Behaviours and Making Decisions Concerning Health
Source: Int J Environ Res Public Health. 2020 Jan 31;17(3):880. doi: 10.3390/ijerph17030880 (PMC7037991; doi:10.3390/ijerph17030880)
Supplement: Supplementary file 1 [file ijerph-17-00880-s001.zip › Appendix 2.pdf]

Appendix 2. Emotions triggered by health or disease information obtained from the Internet*.

	Feeling anxious or fear					Feeling of support or relief				
**Characteristics**	yes		no		p	yes		no		P**
	n	%	n	%		n	%	n	%	
Age groups					0.201					0.348
18-35	92	33.6	182	66.4		70	25.5	205	74.5	
36-59	102	28.3	258	71.7		89	24.7	271	75.3	
60+	34	25.8	98	74.2		25	19.1	106	80.9	
Sex					**0**					**0.021**
Women	152	35.3	278	64.7		117	27.2	313	72.8	
Men	76	22.6	260	77.4		67	19.9	269	80.1	
Education					**0.003**					0.527
Basic/Vocational	44	21.0	166	79.0		44	21.2	164	78.8	
Secondary	94	31.9	201	68.1		74	25.0	222	75.0	
Higher/Some higher	90	34.5	171	65.5		66	25.2	196	74.8	
Employment status					0.244					0.16
Education	15	36.6	26	63.4		8	19.5	33	80.5	
Paid work/ Voluntary/ Other	159	30.6	361	69.4		127	24.4	394	75.6	
Retired/Permanently sick or disabled	40	24.2	125	75.8		34	20.7	130	79.3	
Unemployment	14	35.0	26	65.0		15	37.5	25	62.5	
Residency type					1					**0.044**
Alone	25	29.1	61	70.9		13	15.1	73	84.9	
With family/Other	203	29.9	476	70.1		171	25.2	508	74.8	
Residency place					0.907					0.577
Rural	78	28.7	194	71.3		67	24.6	205	75.4	
Urban	150	30.4	344	69.6		117	23.7	377	76.3	
Mobile use					1					**0.05**
Yes	224	29.8	527	70.2		177	23.5	575	76.5	
No	4	26.7	11	73.3		7	50.0	7	50.0	
Health status					0.587					0.576
Good/very good	144	29.1	350	70.9		125	25.5	366	74.5	
Fair	69	30.1	160	69.9		51	22.0	181	78.0	
Poor/Very poor	14	36.8	24	63.2		8	21.1	30	78.9	
Frequency of Internet use for health purposes					0.051					**0**
Daily	12	36.4	21	63.6		13	39.4	20	60.6	
At least once a month	145	32.9	296	67.1		127	28.9	312	71.1	
At least once a year	59	24.4	183	75.6		37	15.2	207	84.8	

Less than once a year	10	21.3	37	78.7	7	14.6	41	85.4

*Significant differences between groups are marked in bold

** Calculated statistical significance in Fisher's exact independence test
