# Supplementary material for: The Impact of Online Health Information on Patient Health Behaviours and Making Decisions Concerning Health
Source: Int J Environ Res Public Health. 2020 Jan 31;17(3):880. doi: 10.3390/ijerph17030880 (PMC7037991; doi:10.3390/ijerph17030880)
Supplement: Supplementary file 1 [file ijerph-17-00880-s001.zip › Appendix 3.pdf]

Appendix 3. Self-reported pro-health behavior triggered by health or disease information obtained from the Internet*.

	The desire to change diet					The desire to change other habits, ex. quitting smoking, limiting alcohol					Increasing healthy physical activity				
Characteristics	yes		no		P**	yes		no		P**	yes		no		P**
	n	%	n	%		n	%	n	%		n	%	N	%	
Age groups					**0.008**					**0.034**					**0**
18-35	152	55.3	123	44.7		67	24.5	207	75.5		151	54.9	124	45.1	
36-59	170	47.1	191	52.9		60	16.7	300	83.3		167	46.5	192	53.5	
60+	52	39.4	80	60.6		21	16.3	108	83.7		31	23.3	102	76.7	
Sex					**0.029**					**0**					0.189
Women	225	52.2	206	47.8		62	14.5	366	85.5		187	43.4	244	56.6	
Men	149	44.2	188	55.8		86	25.7	249	74.3		162	48.2	174	51.8	
Education					**0.015**					0.456					**0**
Basic/ Vocational	88	41.9	122	58.1		36	17.4	171	82.6		73	34.8	137	65.2	
Secondary	142	47.8	155	52.2		55	18.6	240	81.4		139	47.0	157	53.0	
Higher/ Some higher	144	55.2	117	44.8		57	21.8	204	78.2		137	52.5	124	47.5	
Employment status					0.093					**0.015**					**0**
Education	22	53.7	19	46.3		16	39.0	25	61.0		27	65.9	14	34.1	
Paid work/ Voluntary/ Other	260	49.8	262	50.2		96	18.4	425	81.6		255	49.0	265	51.0	
Retired/ Permanently sick or disabled	68	41.2	97	58.8		27	16.7	135	83.3		46	27.7	120	72.3	
Unemployment	24	60.0	16	40.0		9	23.1	30	76.9		21	52.5	19	47.5	
Residency type					0.734					0.667					**0.026**
Alone	41	46.6	47	53.4		18	20.9	68	79.1		46	51.7	43	48.3	
With family/Other	333	49.0	346	51.0		130	19.2	546	80.8		303	44.8	374	55.2	
Residency place					0.087					**0.028**					**0.026**
Rural	120	44.0	153	56.0		39	14.4	232	85.6		106	39.0	166	61.0	
Urban	254	51.3	241	48.7		109	22.2	383	77.8		243	49.1	252	50.9	
Mobile use					0.44					0.326					1
Yes	365	48.5	388	51.5		147	19.6	602	80.4		342	45.5	410	54.5	
No	9	60.0	6	40.0		1	7.1	13	92.9		7	56.7	8	53.3	
Health status					0.052					0.532					**0**
Good/Very good	256	51.8	238	48.2		94	19.1	397	80.9		260	53.0	231	47.0	
Fair	102	44.2	129	55.8		44	19.2	185	80.8		77	33.0	156	67.0	
Poor/Very poor	14	36.8	24	63.2		10	26.3	28	73.7		11	28.9	27	71.1	
Frequency of Internet use for health purposes					**0**					0.481					**0.009**
Daily	24	72.7	9	27.3		8	24.2	25	75.8		19	57.6	14	42.4	
At least once a month	235	53.3	206	46.7		90	20.5	348	79.5		216	49.1	224	50.9	
At least once a year	103	42.4	140	57.6		44	18.3	197	81.7		98	40.3	145	59.7	
Less than once a year	11	22.9	37	77.1		6	12.5	42	87.5		14	29.8	33	70.2	

*Significant differences between groups are marked in bold

** Calculated statistical significance in Fisher's exact independence test
