# Supplementary material for: The Impact of Online Health Information on Patient Health Behaviours and Making Decisions Concerning Health
Source: Int J Environ Res Public Health. 2020 Jan 31;17(3):880. doi: 10.3390/ijerph17030880 (PMC7037991; doi:10.3390/ijerph17030880)
Supplement: Supplementary file 1 [file ijerph-17-00880-s001.zip › Appendix 4.pdf]

Appendix 4. Self- reported health decisions triggered by health or disease information obtained from the Internet*

Part A

	Asking the doctor for suggestions or questions about the diagnosis or treatment of diseases					Asking the doctor for referral / additional screening tests					Taking the drug or changing the medication without consulting a doctor				
Characteristics	yes		no		P**	yes		no		P**	yes		no		P**
	n	%	n	%		n	%	n	%		n	%	n	%	
Age groups					0.202					0.257					0.942
18-35	105	38.2	170	61.8		88	32.0	187	68.0		23	8.4	252	91.6	
36-59	156	43.2	205	56.8		137	37.8	225	62.2		33	9.1	330	90.9	
60+	46	35.1	85	64.9		42	32.3	88	67.7		12	9.1	120	90.9	
Sex					0.374					0.253					0.61
Women	179	41.4	253	58.6		157	36.6	272	63.4		36	8.3	396	91.7	
Men	128	38.2	207	61.8		110	32.5	228	67.5		32	9.5	306	90.5	
Education					0.807					0.882					0.422
Basic/vocational	87	41.8	121	58.2		70	33.5	139	66.5		23	11.0	187	89.0	
Secondary	115	38.9	181	61.1		103	34.9	192	65.1		25	8.4	272	91.6	
Higher/some higher	105	39.9	158	60.1		94	35.7	169	64.3		20	7.6	243	92.4	
Employment status					0.341					0.759					0.257
Education	11	26.8	30	73.2		11	26.8	30	73.2		7	17.1	34	82.9	
Paid work/ voluntary/ other	216	41.3	307	58.7		185	35.4	337	64.6		42	8.0	481	92.0	
Retired/ permanently sick or disabled	65	39.6	99	60.4		57	34.8	107	65.2		16	9.6	150	90.4	
Unemployment	15	38.5	24	61.5		14	35.0	26	65.0		3	7.5	37	92.5	
Residency type					0.419					0.408					0.844
Alone	39	44.3	49	55.7		27	30.7	61	69.3		8	9.1	80	90.9	
With family/other	268	39.5	410	60.5		240	35.4	438	64.6		60	8.8	621	91.2	
Residency place					0.847					0.547					0.747
Rural	109	40.1	163	59.9		99	36.5	172	63.5		27	9.9	246	90.1	
Urban	198	40.0	297	60.0		168	33.9	328	66.1		41	8.2	456	91.8	
Mobile use					0.095					0.575					1
Yes	298	39.6	455	60.4		261	34.7	492	65.3		67	8.9	688	91.1	
No	9	64.3	5	35.7		6	42.9	8	57.1		1	6.7	14	93.3	
Health status					0.133					0.065					0.201
Good/very good	192	38.7	304	61.3		165	33.3	330	66.7		7	21.2	26	78.8	

Fair	92	40.4	136	59.6		81	35.1	150	64.9		39	8.8	404	91.2	
Poor/very poor	21	55.3	17	44.7		19	52.8	17	47.2		18	7.4	225	92.6	
Frequency of Internet use for health purposes					**0**					**0**					0.098
Daily	19	57.6	14	42.4		17	53.1	15	46.9		7	21.2	26	78.8	
At least once a month	202	45.6	241	54.4		182	41.4	258	58.6		39	8.8	404	91.2	
At least once a year	73	30.2	169	69.8		59	24.3	184	75.7		18	7.4	225	92.6	
Less than once a year	12	26.1	34	73.9		8	16.7	40	83.3		3	6.2	45	93.8	

*Significant differences between groups are marked in bold

** Calculated statistical significance in Fisher's exact independence test

Part B

	Making an appointment with a doctor					Cancellation of a doctor's appointment					Resignation from planned tests or medicines used				
Characteristics	yes		no		p	yes		no		p	yes		no		p
	n	%	n	%		n	%	n	%		n	%	n	%	
Age groups					0.259					0.186					0.946
18-35	132	48.0	143	52.0		62	22.5	213	77.5		19	6.9	256	93.1	
36-59	162	44.8	200	55.2		85	23.4	278	76.6		26	7.2	335	92.8	
60+	52	39.4	80	60.6		21	15.9	111	84.1		8	6.1	123	93.9	
Sex					0.771					0.539					0.085
Women	196	45.5	235	54.5		98	22.7	334	77.3		36	8.4	395	91.6	
Men	150	44.4	188	55.6		70	20.7	268	79.3		17	5.1	319	94.9	
Education					**0.007**					0.2					0.321
Basic/vocational	81	38.8	128	61.2		49	23.3	161	76.7		19	9.1	189	90.9	
Secondary	127	42.8	170	57.2		55	18.5	242	81.5		17	5.7	280	94.3	
Higher/some higher	138	52.5	125	47.5		64	24.3	199	75.7		17	6.5	245	93.5	
Employment status					0.25					**0.022**					0.691
Education	17	41.5	24	58.5		10	24.4	31	75.6		4	9.8	37	90.2	
Paid work/ voluntary/ other	248	47.4	275	52.6		117	22.4	406	77.6		37	7.1	483	92.9	
Retired/ permanently sick or disabled	64	38.8	101	61.2		26	15.7	140	84.3		9	5.4	157	94.6	
Unemployment	17	42.5	23	57.5		15	37.5	25	62.5		3	7.5	37	92.5	
Residency type					0.91					0.054					0.656
Alone	39	44.3	49	55.7		12	13.6	76	86.4		7	8.0	81	92.0	

With family/other	307	45.1	373	54.9		156	22.9	525	77.1		46	6.8	632	93.2	
Residency place					**0.05**					0.262					0.806
Rural	109	40.1	163	59.9		52	19.0	221	81.0		21	7.7	251	92.3	
Urban	237	47.7	260	52.3		116	23.3	381	76.7		32	6.5	463	93.5	
Mobile use					1					0.109					**0.016**
Yes	339	45.0	415	55.0		162	21.5	593	78.5		49	6.5	703	93.5	
No	7	46.7	8	53.3		6	40.0	9	60.0		4	26.7	11	73.3	
Health status					0.926					0.782					0.742
Good/very good	223	45.0	273	55.0		111	22.4	385	77.6		36	7.3	460	92.7	
Fair	104	45.0	127	55.0		47	20.3	184	79.7		16	7.0	214	93.0	
Poor/very poor	18	48.6	19	51.4		9	23.7	29	76.3		1	2.8	35	97.2	
Frequency of Internet use for health purposes					**0.007**					**0.02**					**0.005**
Daily	19	57.6	14	42.4		12	36.4	21	63.6		5	15.6	27	84.4	
At least once a month	215	48.6	227	51.4		107	24.2	336	75.8		37	8.4	404	91.6	
At least once a year	97	39.9	146	60.1		40	16.5	203	83.5		7	2.9	236	97.1	
Less than once a year	14	29.2	34	70.8		9	18.8	39	81.2		3	6.2	45	93.8	

*Significant differences between groups are marked in bold

** Calculated statistical significance in Fisher's exact independence test
