# Supplementary material for: The Impact of Online Health Information on Patient Health Behaviours and Making Decisions Concerning Health
Source: Int J Environ Res Public Health. 2020 Jan 31;17(3):880. doi: 10.3390/ijerph17030880 (PMC7037991; doi:10.3390/ijerph17030880)
Supplement: Supplementary file 1 [file ijerph-17-00880-s001.zip › questionnaire.pdf]

Appendix: E-Health consumer trend survey 2017

QUESTIONNAIRE

QTS1 Interviewer number
Interviewer number.........................1
QTS2 Start time
Start time...1
QTS3 Date
Date...1
POST
Postal code.......................................1

Q1
Good Morning/good afternoon/good evening, my name is ______________________________, and I am phoning from __________________ on behalf of Wroclaw Medical University. We are conducting a national survey about use of the Internet and other sources for health information. In this connection we would like to speak to a person who is at least 18 years old.

Everyone who takes part in the survey is completely anonymous (we would like to emphasize that there are no right or wrong answers). The interview will take about 15 minutes and your answers will be very valuable to us.
Would you be prepared to take part?

REGISTER GENDER

Male 1

Female..2

Q2
A few introductory questions.
How old are you?

Age:...1

DK/NA 99

Q3

What is your highest level of education completed?

- BASIC SCHOOL NONE...01
- BASIC PROFESSIONAL..02
- SECONDARY...03
- HIGHER (Bachelor, Master, Engineer)..04

- SOME HIGHER …………………………………………………………..……………..05
- Do not want to answer ………………………….…………………………06

DK/NA 99

Q4
Do you live with family or alone?
Alone ……………………………………………………………1
With family……………………………………………………2
Other (e.g. roommate)…………………………………3
DK/NA 9

Q5
Where do you live?
- **City** (main cities)………………………………………………………………………1
- **Minor cities** (suburbs/vicinity to larger cities)……………………………2
- **Villages** ……………………………………………………………………………………3
- **Rural area** (country-side, scattered population) …………………………4

Q6
Which of these descriptions best describes your situation or applied to what you have been doing for the last month: READ OUT

- Paid work (including self-employed ……………………………………………..1
- In education………………………………………………………………………………2
- Unemployed……………………………….……………………………………………3
- Permanently sick or disabled…………………………………………….……………4
- Retired ……………………………………………………………………………………..5
- In community or military service…………………………………………………….6
- Housework, looking after children or other persons (e.g. maternity leave)7
- (Other)………………………………..…………………………………………………….8

  DK/NA 9

Q7
I will now read a list of various sources of information about health or illness, and would like to know how important these are to you. Please would you answer on a scale from 1 to 5, where 1 is "not important" and 5 is "very important".
READ OUT:
1 Not important 2 3 4 5 Very important 9 DK/NA

Internet……………………………………………………………………………….**1 2 3 4 5 9** 1
TV/radio………………………………………………………………………………..**1 2 3 4 5 9** 2
Books, medical encyclopedias and leaflets…………………………………….**1 2 3 4 5 9** 3
Courses and lectures ………………………………………………………………..**1 2 3 4 5 9** 4
Newspapers, magazines …………………………………………………………….**1 2 3 4 5 9** 5

Family, friends and colleagues……………………………………………………………**1 2 3 4 5 9** 6
Pharmacies……………………………………………………………………………..……..**1 2 3 4 5 9** 7
Direct face-to-face contact with health professionals …………………**1 2 3 4 5 9** 8

Q8. How often do you use the Internet?

READ OUT

- Every day …………………………………………………………..…………1
- Every week…………………………………………………………………………..2
- Every month…………………………………………………………………………3
- Less than once a month…………………………………………………4
- **I have never used the Internet (→ Q15)**………………… 5
- I have never used it, but I have asked others to use it for me….6

DK/NA9

Q9. How often do you use the Internet to get information about health or illness? READ OUT

Every day ……………………………………………………………………1
Every week ……………………………………………………………………2
Every month ………………………………………………………………3
Every six months …………………………………………………4
Every year ………………………………………………………………5
Less than once a year………………………………………………6
Never **(→ Q15)……………………………………………………7**
DK/NA9

Q10 I will now read out some purposes for which the Internet can be used to provide information related to health or illness, and would like to know how often you use the Internet for these purposes:

[REPEAT SCALE WHEN NECESSARY]

□ Every day	1
□ Every week	2
□ Every month	3
□ Every six months	4
□ Every year	5
□ Less than once a year	6
□ Never	7

DK/NA9

How often do you use the Internet to:
a. interact with health professionals you have not met face-to-face………………………………………………………………….**12345679**1
b. participate In forum or self help groups (focusing on health or illness)………………………………………………………………. **12345679**2
c. order medicines or other products related to health or illness management online………………………………………………………………………**12345679**3
d. read about health and illness………………………………**12345679**4

Yes (→ Q10B)..1
No (→ Q11)...2

Q10B. If you wanted to find out about health or illness via the Internet, what kind of information did you search on the Internet?

- general information on a healthy lifestyle 1
- information on proper nutrition, e.g. cholesterol-restricted diets, slimming diets, diets rich in vitamins and microelements, etc. 2
- information on healthy forms of physical activity (e.g. running, aerobics, cycling, swimming, etc.) 3
- information related to the proper development of pregnancy and the child in different periods of life 4
- information on mandatory and recommended preventive vaccinations 5
- information about various diseases 6
- information on dealing with various addictions, e.g. smoking, alcohol abuse 7
- information about various medicines, herbs and supplements 8
- information on doctors or other professional medical staff or medical facilities / health centers 9
- other 10

Q11. Have you approached your family doctor, specialist, or other health professional(s) over the Internet (Web or e-mail), e.g. read their website, request or renew prescription, schedule an appointment, ask particular health questions or read your health record?

Yes (→ Q12) 1
No (→ Q13) 2
DK/NA 9

FILTER Q11 = 1

Q12. In which connection and for what purposes have you approached your family doctor, specialist, or other health professional(s) via the Internet?
READ OUT. CHECK AS MANY AS APPLY.

- Request or renew prescription via e-mail or Web 1
- Schedule an appointment...2
- Ask particular health question...3
- Access to read your patient record 4
- Read their website...5
- Other...6

DO NOT READ OUT
Do not want to answer...8

DO NOT READ OUT
Do not know...9

Finish Filter Q11=1
Filter Q11=2

Q13. There are different reasons for not approaching your family doctor, specialist or other health professional(s)via the Internet. Which reasons apply to you?
READ OUT. CHECK AS MANY AS APPLY.

- I worry about confidentiality……………………………………..1
- I prefer face-to-face communication………………..………..2
- My family doctor or specialist do not offer such services………..3
- I have not needed to contact them…………………………………………4
- I would, but I cannot use the internet (I do not have sufficient skills in this area)……………………………………………………………………………………….. 5
- Other …………………………………………………………………………………6

DO NOT READ OUT
- Do not want to answer …………………………………………8

DO NOT READ OUT
- Do not want …………………………………………………………….9

Finish filter: Q11=2

Q14. Has information on health or illness which you have obtained from the Internet led to any of the following?

READ OUT YES (1) NO (2) DO NOT KNOW (9)

A.

- Feelings of anxiety or fear…………………………………………………………**129**1
- Feelings of spiritual support or relief ………………………………………….**129**2
- Desire to change diet ……………………………………. …………………**129**3
- Desire to change other habits, ex. quitting smoking, limiting alcohol…**129**4
- Increasing healthy physical activity ……………………………………………..**129**5

B.
- Asking the doctor for suggestions or questions about the diagnosis or treatment of diseases ……………………………………………………..**129**6
- Asking the doctor for referral / additional screening tests………………..**129**7
- Taking the drug or changing the medication without consulting a doctor…………………………………………………………………………………..**129**8
- Resignation from planned tests or medicines used……………………..**129**9
- Making an appointment with a doctor……………………………..…….**129**10
- Cancellation of a doctor's appointment…………………………………….**129**11

Q15. If you were to find a new doctor, state the importance of the following factor for your decision. Please would you answer on a scale from 1 - "not important" to 5 – "very important"
1 Not important 2 3 4 5 Very important 9 do not know / not applicable

- The possibility of receiving simple medical recommendations via SMS or e-mail ……………………………………………………………………………**123459**1
- The possibility to prescribe medicines via e-mail or the Internet……………………………………………………………….…. **123459**2
- The possibility of setting or changing the date of the visit via the Internet ……………………………………………………………………………..**123459**3

- Information about medical practice on the Internet (certificates, practice position among other medical facilities)…………………… …………**123459**4
- The doctor's office has its own website………………………………**123459**5
- The possibility of contact via e-mail……………………………………….**123459**6
- The possibility to receive SMS notifications…………………………..**123459**7
- Online access to the patient's medical records………………………**123459**8

Q16. I will now read two statements for you and I will ask you to tell me, which of the statements you agree most with:

A: "I do not feel comfortable to have a health visit via a computer or a video-phone"

B: "I am positive to the idea of having a health visit via a computer or a video phone"

Which statement do you agree most with?

□ I mostly agree with statement A 1
□ I mostly agree with statement B 2
□ I do not know **[do not read this option]** 9

Q16.1 Would you agree to pay 10 € for such a visit?
□ Yes 1
□ No 2
□ YES - through private insurance **[do not read]** 11
□ YES **–** through public insurance **[do not read]** 12

Q17. I will now read two statements for you and ask you to tell me, which you agree most with:

A: "In order to get a quick and valid diagnosis, I am positive about giving internet access to my medical record to a doctor in another location or abroad, e.g. to give consultation about an MRI."
B: "Even if I were to receive a quick and accurate diagnosis, I do not feel comfortable providing access to my medical record to a doctor in another location or abroad, e.g. to give consultation about an MRI. "

Which statement do you agree most with?
□ I mostly agree with statement A 1
□ I mostly agree with statement B 2
□ I do not know **[do not read this option]** 9

Q 18. Assuming that you had the possibility to use the internet to access your electronic health record online, would you do it?
□ Yes 1
□ No 2

□ I do not know **[do not read this option]** 9

Q18.1 Would you agree to pay 30 € per year for this service?
□ Yes 1
□ No 2
□ YES, through private insurance **[do not read]** 11
□ YES, through public insurance **[do not read]** 12

Q 19.
Do you have your own mobile phone?

- Yes 1
- Yes, but I can use it only in limited scope (e.g. only phone calls, I cannot use SMS) 2
- No 9

Yes → Q19.1
No → Q20

Q19.1 Which of telemedicine services in particular would you like to use?

CHECK AS MANY AS APPLY.

- SMS reminders of planned visits or prescribed drugs………………1
- teleconsultation via the personal computer or mobile-phone…..2
- remote monitoring of basic health measurements, e.g. blood pressure, ECG, heart and lung auscultation, blood glucose concentrations, weight, temperature etc………………………..3
- remote/online reporting about medical test results……………4
- receiving simple medical recommendations directly via a mobile phone or computer…………………………………………………………5
- other (what?)…………………………………………………………… 6

To end off, a few background questions:

Q20. How many times did you visit the doctor during last 12 months (include hospitalization or visits to the outpatient department; do NOT include visits to the dentist)?
NA998/ DK 999

Number of times………………………………………………………………………………………1

Q21. Are you, or someone close to you, currently experiencing long-term illness or disability?
CHECK AS MANY AS APPLY

- Yes I am…………………………………………………1

- Yes, someone close……………………………….2
- No ……………………………………………………….3
- **DO NOT READ OUT**

- Do not want to answer………………………….8
- **DO NOT READ OUT**

- Do not know ……………………………………….9

Q22. How would you assess your present state of health ?
READ OUT

- Very good……………………………………………1
- Good…………………………………………………….2
- Fair……………………………………………………….3
- Bad……………………………………………………….4
- Very bad …………………………………………….5
- **DO NOT READ OUT**
- Do not want to answer …………………………8
- **DO NOT READ OUT**
- Do not know ……………………………………….9

Thank you very much for your help!
